# Exosome-derived CIRP: An amplifier of inflammatory diseases

**DOI:** 10.3389/fimmu.2023.1066721

**Published:** 2023-02-14

**Authors:** Jingrun Han, Yibo Zhang, Peng Ge, Tikam Chand Dakal, Haiyun Wen, Shuangfeng Tang, Yalan Luo, Qi Yang, Bianca Hua, Guixin Zhang, Hailong Chen, Caiming Xu

**Affiliations:** ^1^ Department of General Surgery, The First Affiliated Hospital of Dalian Medical University, Dalian, Liaoning, China; ^2^ Institute (College) of Integrative Medicine, Dalian Medical University, Dalian, Liaoning, China; ^3^ Laboratory of Integrative Medicine, The First Affiliated Hospital of Dalian Medical University, Dalian, Liaoning, China; ^4^ Genome and Computational Biology Lab, Mohanlal Sukhadia University, Udaipur, Rajasthan, India; ^5^ Department of Molecular Diagnostics and Experimental Therapeutics, Beckman Research Institute of City of Hope, Biomedical Research Center, Comprehensive Cancer Center, Monrovia, CA, United States

**Keywords:** cold-inducible RNA-binding protein, exosome, immune response, inflammation, targeted therapy

## Abstract

Cold-inducible RNA-binding protein (CIRP) is an intracellular stress-response protein and a type of damage-associated molecular pattern (DAMP) that responds to various stress stimulus by altering its expression and mRNA stability. Upon exposure to ultraviolet (UV) light or low temperature, CIRP get translocated from the nucleus to the cytoplasm through methylation modification and stored in stress granules (SG). During exosome biogenesis, which involves formation of endosomes from the cell membrane through endocytosis, CIRP also gets packaged within the endosomes along with DNA, and RNA and other proteins. Subsequently, intraluminal vesicles (ILVs) are formed following the inward budding of the endosomal membrane, turning the endosomes into multi-vesicle bodies (MVBs). Finally, the MVBs fuse with the cell membrane to form exosomes. As a result, CIRP can also be secreted out of cells through the lysosomal pathway as Extracellular CIRP (eCIRP). Extracellular CIRP (eCIRP) is implicated in various conditions, including sepsis, ischemia-reperfusion damage, lung injury, and neuroinflammation, through the release of exosomes. In addition, CIRP interacts with TLR4, TREM-1, and IL-6R, and therefore are involved in triggering immune and inflammatory responses. Accordingly, eCIRP has been studied as potential novel targets for disease therapy. C23 and M3, polypeptides that oppose eCIRP binding to its receptors, are beneficial in numerous inflammatory illnesses. Some natural molecules such as Luteolin and Emodin can also antagonize CIRP, which play roles similar to C23 in inflammatory responses and inhibit macrophage-mediated inflammation. This review aims to provide a better understanding on CIRP translocation and secretion from the nucleus to the extracellular space and the mechanisms and inhibitory roles of eCIRP in diverse inflammatory illnesses.

## Graphical Abstract

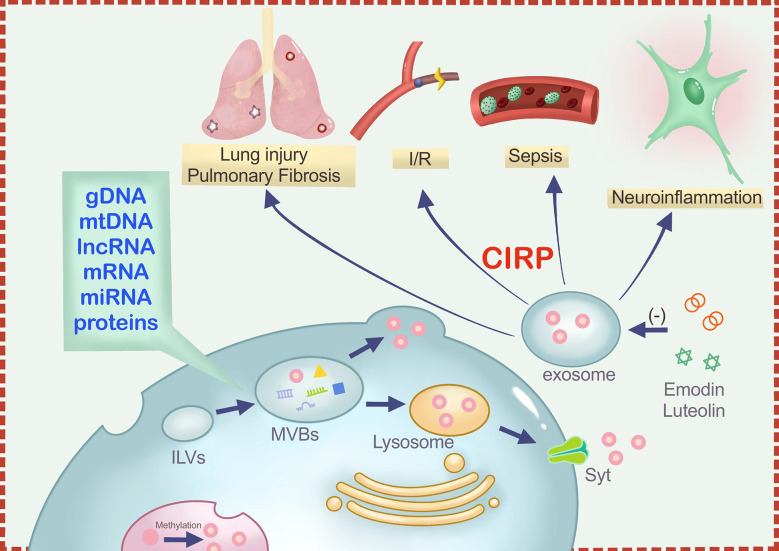

## 1 Introduction

Cold-inducible RNA-binding protein (CIRP) were first discovered in mouse testis more than two decades ago (1). It came to the attention of researchers as a result of its expression during moderate cold stress. The RG/RGG region of CIRP is critical for mediating CIRP phase separation *in vitro* and stress granules (SGs) association in cells (2). As a RNA-binding protein (RBP), CIRP plays important roles in transcription, pre-mRNA processing/transport, mRNA degradation, translation, and non-coding RNA processing. Studies have shown that the 3`-UTR binding sites of CIRP are enriched within 100 nucleotides upstream of the polyadenylation sites, and UU and UUU are most likely the core recognition sequences of CIRP (3). CIRP consists of an N-terminal RNA-recognition motif (RRM) and a C-terminal arginine-rich region. The X-ray quaternary structure of the CIRP RRM has been resolved recently and four important residues with possible involvement in protein-nucleic acid binding have been identified (4). Expression of CIRP during cold stress modulates RNA stability and translation in hibernating mammals that reduce their body temperature from 37°C to as low as 0~5°C during bouts of dullness (5). CIRP is translocated from the nucleus to the cytoplasm, whereby they stabilize mRNAs. They can regulate mRNA transcripts and protect them from degradation for future protein synthesis. Studies have also shown that the expression of CIRP can also be regulated by several kinds of stress conditions suggesting that CIRP is generally expressed as stress-response proteins. Besides, CIRP is widely expressed in a large variety of tissues and cells.

According to the literature, iCIRP has been implicated in multiple cellular processes such as cell proliferation (6), cell survival (7, 8), telomere maintenance (9), circadian modulation (10), DNA repair (11) and tumor formation and progression (12, 13). Intracellular CIRP (iCIRP) can migrate from the nucleus to the cytoplasm *via* methylation-dependent mechanism in response to stress and regulate the stability of mRNA through their binding sites on the 3′-UTR of their target mRNAs (3). The translocation of CIRP is dependent on GSK3β and CK2. Both GSK3β and CK2 cause phosphorylation of CIRP and affect its cellular localization and pretreatment of cells with either CK2 inhibitors or GSK3β inhibitors prior to UV treatment significantly reduce CIRP localization to the cytosol (14). These results suggest that both methylation and phosphorylation of CIRP is required for CIRP translocation to the cytosol upon stress stimulus. Some studies have demonstrated that both CK2 sites and GS3Kβ sites on CIRP have overlapping functional role in regulating CIRP translocation to the cytosol, but only GSK3β sites are involved in the RNA-binding activity of CIRP, in response to UV radiation (14, 15).

In contrast to its functions in the intracellular space, extracellular CIRP (eCIRP) has been discovered recently as a damage-associated molecular pattern (DAMP) capable of triggering inflammation in various inflammatory conditions (16), including sepsis (17), neuroinflammation (18)and ischemia-reperfusion injury (19–24). For example, administering exogenous CIRP to healthy mice was shown to induce lung injury through vascular leakage, neutrophil infiltration, local production of pro-inflammatory cytokines, and activating the NLRP3 inflammasome in the vascular endothelial cells of the lung (25). Altogether, a fair number of studies have investigated the potential role of eCIRP in different models of inflammatory diseases, such as sepsis (16, 26–29), acute pancreatitis (30), and Alzheimer’s disease (31). The important pro-inflammatory role of eCIRP suggests that targeting eCIRP may be of potential therapeutic importance in controlling inflammatory diseases. Nevertheless, the translation of such findings into clinical practice still remains nascent field to be explored.

Here, we summarize how eCIRP, especially exosome derived CIRP, amplify inflammation in different inflammatory conditions with the aim of providing new insights for the development of novel targeted therapies.

## 2 Conventional secretion mechanisms of eCIRP

CIRP release mechanisms include necrosis, lysosome-mediated release, extracellular traps, and exosomes (32).CIRP can be released either *via* inflammasome activation or passively following cell death (33). Necroptosis, apoptosis, pyroptosis, and ferroptosis might contribute to the passive release of the CIRP (32). Studies have shown that CIRP is among the main factors that induces mitochondrial DNA (mtDNA) fragmentation in damaged tissues (34). However, according to an *in-vitro* study on macrophages, it has been shown that cell necrosis does not trigger the passive release of CIRP (16).

Active release of CIRP is mediated mainly by lysosomes and exosomes. When cells are stimulated by oxidative stress, hypothermia, ultraviolet (UV) radiation, etc. CIRP are transferred to the extracellular space through the lysosomal pathway (12, 33). CIRP migrates from the nucleus to cytoplasmic SGs *via* a methylation-dependent mechanism and act as translational repressors. Stressors such as oxidative stress, endoplasmic reticulum (ER) stress, osmotic shock, and heat shock may lead to the methylation of iCIRP (35). Oxidative stress leads to CIRP migration to SGs without altering their expression (36). SGs are dynamic cytoplasmic foci in which stalled translation initiation complexes accumulate in cells that are subjected to environmental stress. The relocation of CIRP to the SG also occurs in osmotic stress, heat shock and in response to endoplasmic reticulum stress (36). CIRP/hnRNP A18 is an RNA-binding factor consisting of an RNA recognition motif (RRM) at the N-terminus and a region at the c-terminus containing multiple repeats of the RGG motif. Methylation is important for the recruitment of CIRP from the nucleus to the SGs. As mentioned earlier, TIA-1 has been shown to be the main mediator of SGs formation (37). In the absence of TIA-1 or TIAR proteins, CIRP is still recruited to SGs. Since CIRP does not contain any signaling peptides, their secretion cannot be mediated by the classical Endoplasmic Reticulum-Golgi-dependent pathway. In one study, biochemical fractionation revealed that CIRP is enriched in the lysosomal compartment of macrophages undergoing hypoxia, suggesting that CIRP is released through lysosomal secretions (16, 35). As a class of DAMPs, eCIRP conforms to the general mechanism of the extracellular release of DAMPs (32). The well-studied carriers of DAMPs during active release are the secreted lysosomes and exosomes, both of which are normally secreted by exocytosis (16, 38).

Lysosomal secretion is a typical feature of stressed cells and has been also demonstrated to be a release pathway for eCIRP (16, 32). The synaptotagmin (Syt) assay found that lysosomal secretion can be initiated by stimulating cell surface receptors as well as through increasing intracellular Ca^2+^. Syt mobilizes lysosomes to the microtubule organizing center, where lysosomes are associated with kinesin motility (39). The motor proteins further transport the lysosome near the secretion site, where the lysosome travels to the docking site using actin-based movements. The docking and fusion of lysosomes with the plasma membrane are mediated by Rabs and SNARE (Soluble NSF Attachment Protein Receptors) complexes, respectively (39, 40).

## 3 Regulation of CIRP expression

The overexpression of CIRP has been consistently witnessed in different organs and species (from amphibians to humans) upon mild hypothermia or cold stress (10), which supports the plausible protective role of CIRP in adaptation of these species to cold stress. However, CIRP homologs in different species responded differently to different environmental stresses. Besides this, CIRP is also overexpressed upon UV radiation, mild hypoxia and low glucose conditions, but is under expressed in response to heat stress stimulus and upon treatment with inflammatory cytokines such as TNF-α and TGF-β (12), underscoring that CIRP is general stress responsive protein. While, CIRP is overexpressed upon mild hypoxia, the chronic hypoxia results in completely opposite genetic outcome, that is low CIRP expression (13). Glycogen synthase kinase 3β (GSK3β) is an important serine/threonine kinase, which is involved in various biochemical processes in cells, for instance cell metabolism, cell cycle, transcription, vesicular transport and others (41). Activation of GSK3β has shown to increase the transcription of CIRP (16). CIRP is also upregulated by IGF1 (17). As expected, the transcription of the CIRP homolog in Salmon can be upregulated by osmotic stress, but not by cold stress (18) as expected to be respond differently in a species-specific manner. Similar to this, there exist different homologs of CIRP in Xenopus cells including xCIRP, xCIRP-1 and xCIRP-2. All homologs express and respond differently to different environmental stimulus. For instance, low temperature (cold stress) induces overexpression of xCIRP and xCIRP 2, but not xCIRP-1 (9, 19, 20). This implies that the CIRP is a general stress protein and its expression and response to a particular stress differs significantly between species.

## 4 Exosomal CIRP

An essential mode of intercellular communication is the transport of information components such as proteins, nucleic acids, and lipids *via* exosomes (42, 43), extracellular vesicles measuring between 40 and 100 nm in diameter and found in various bodily fluids (44). Exosomes support the interior environment’s balance under normal physiological settings (45). However, these exosomes may have radically altered contents in a state of excessive inflammation. Many studies have shown that miRNAs such as miR-155 (46) and miR-21 (47) transported by exosomes play a crucial role in inflammation. It has been shown that CIRs can be mainly released outside the cell in two ways: passively through necrosis and actively through lysosomes (32). However, Murao et al. (48), discovered that CIRP might stimulate inflammatory cell aggregation and activation through the exosome secretion.

**Figure 1:**
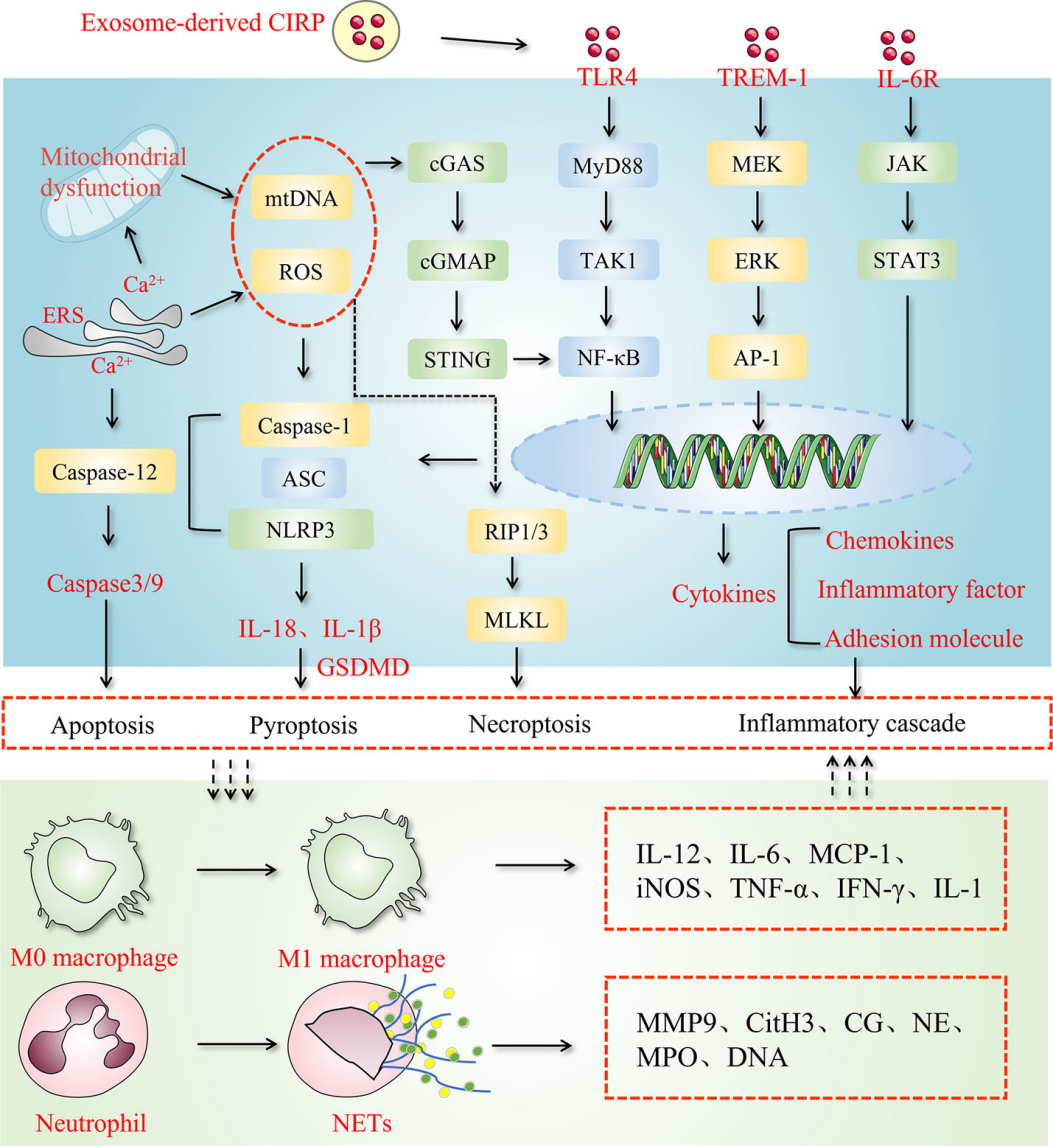
Exosome-derived CIRP: an amplifier of inflammatory cascades.

Murao and colleagues found that the serum exosomes of LPS-injected and CLP-operated mice contained significantly higher amounts of eCIRP than those in control animals. Similarly, activation of macrophages with LPS resulted in a significant increase in the release of exosomal CIRP. Based on these findings, it appears that exosome-carried CIRP are a substantial source of eCIRP. Accordingly, the presence of eCIRP in exosomes could be justified by the presence of the lipid bilayer on the surface of exosomes, which can protect proteins or microRNAs from being degraded during transport process. CD63 is a well-known marker used to identify exosomes (49). Studies have revealed that CIRP can stably bind to CD63. Furthermore, they discovered that exosome biogenesis and release inhibitors (50) could not only block exosome release but also simultaneously decrease the expression of eCIRP. With the exposure of inflammatory cells to exosomes, eCIRP present on the surface of the exosomes stimulates the release of pro-inflammatory cytokines and migration of neutrophils by binding to cell surface receptors (48) (**Figure 1**). However, there is no direct evidence of eCIRP release from the interior of exosome/luminal vesicles to the surface. Through literature review, it was found that there were transmembrane proteins on the exosome surface, such as lysosome-associated membrane protein (LAMP) and transferrin receptor (TfR) (51).Therefore, we hypothesized that proteins carried in exosomes cross the phospholipid bimolecular wind of exosomes through the stimulation of specific signaling molecules to reach the surface, and CIRP is no exception. Taken together, these findings support the notion that exosomes are mediators of CIRP release that targeting exosomal CIRP may represent a novel treatment strategy.

## 5 Role of eCIRP in inflammatory diseases

In inflammatory diseases, eCIRP acts as an inflammatory amplifier by binding to its receptors. TLR4, TREM-1, and IL-6R are three receptors to which CIRP binding occurs, as mentioned in the **Table 1**. TLR4 is a member of an important class of molecules involved in adaptive immunity and acts as a bridge between adaptive and innate immunity. CIRP binds to TLR4-MD2, a complex formed in systemic inflammation induced by hemorrhagic shock and sepsis (16). Hemorrhagic shock increases eCIRP levels and activates STING through the TLR4/MyD88/TRIF pathway to exacerbate inflammation (55). In lung fibroblasts, eCIRP amplifies pro-inflammatory cytokines in a TLR4-dependent manner, triggering pulmonary fibrosis (54). As a receptor for eCIRP, TREM-1 plays a vital role in ischemia-reperfusion in the liver, intestine, and other organs (60), and induces inflammation in macrophages and neutrophils (35), forming NETs (61). In hemorrhagic shock and sepsis, TREM-1 can also be a key target of eCIRP (16, 56, 62). In the alveoli, TREM-1 can act as a target to trigger inflammation in alveolar type II cells (57). One study showed a strong binding affinity between eCIRP and IL-6R (58). Supporting this finding, it is well known that CIRP can skew the macrophages towards the M2 phenotype and that using IL-6R antibodies CIRP can inhibit M2 polarization (58). In neurological diseases, eCIRP activates the IL-6Rα/STAT3/Cdk5 pathway in neurons, inducing neuroinflammation (59). Combining the above two studies, we can find that IL-6R plays a double-edged sword role in inflammation. It can improve the tolerance of macrophages to endotoxin in sepsis, and can also promote microglial inflammation in the nervous system.

**Table 1:** eCIRP-related receptors and their biological functions.

Receptor	Studied model	Organ	Biological function	References
TLR4	Cecal ligation and puncture (CLP)	Lung	Induces endoplasmic reticulum stress	(52)
	CLP	Heart and liver	Promotes macrophage inflammation	(16)
	Pseudo fracture trauma	–	Triggers macrophage cell death	(34)
	Ischemia-reperfusion injury	Liver	Mediates mitochondrial fission, inflammatory responses, and apoptosis.	(53)
	Bleomycin-induced pulmonary fibrosis	Lung	Promotes proinflammatory pulmonary fibroblast phenotype	(54)
	Hemorrhagic shock	Lung	Induces inflammation and tissue damage	(55)
TREM-1	CLP	Lung	Induces systemic inflammation and lung injury	(56)
	–	Lung	Promotes alveolar type II cells inflammation	(57)
IL-6R	CLP	–	Promotes macrophage endotoxin tolerance	(58)
	Amyloid β (Aβ)-mediated microglial inflammation	Neurons	Upregulation of neuronal inflammation	(59)

### 5.1 Sepsis

Sepsis is a life-threatening inflammatory disorder caused by a dysregulated host immune response to infection (63). In a recent survey, the annual global incidence of sepsis has been estimated at 31.5 million cases resulting in 5.3 million deaths worldwide every year (64). According to a previous study (16), CIRP translocates from the nucleus to the cytoplasm and are subsequently released into the circulation during sepsis (33). As a well-described member of the DAMP family, there is evidence that eCIRP plays a crucial role in regulating sepsis. eCIRP has long been the subject of great interest in sepsis.

In this context, the regulatory effects of eCIRP on neutrophils has received extensive attention due to the vital roles of neutrophils in sepsis. For instance, ICAM-1-positive neutrophils, *via* increased production of neutrophil extracellular traps (NETs) and inducible nitric oxide synthase (iNOS), play a major role in the induction of exaggerated inflammation during sepsis (65). Furthermore, eCIRP has been shown to promote the formation of NETs by inducing PAD4 expression in the lungs of a mouse model of sepsis (66). By activating TREM-1 on the surface of neutrophils, eCIRP induces NET-forming ICAM-1-positive neutrophils (61). In addition, ICAM-1-mediated Rho activation further promotes the formation of NETs as a novel pathway (61). Another study showed that eCIRP could induce neutrophil reverse transendothelial migration (rTEM) in sepsis by increasing neutrophil elastase (NE) and decreasing the junctional adhesion molecule-C (JAM-C) (27). The reversely migrated (RM) neutrophils showed a prolonged lifespan and were associated with systemic inflammation in the cremaster muscle ischemia-reperfusion (I/R) injury in a mouse model (67). These studies consistently indicate that RM neutrophils may contribute to the exacerbation of a local inflammation into a systemic inflammatory status. CIRP also triggered endoplasmic reticulum (ER) stress *via* TLR4 activation in preclinical models of sepsis and promoted inflammation, apoptosis, and histological injury (52). Induction of ER stress due to CIRP release in sepsis can be blocked by creating knockout of TLR4 or CIRP in mice, suggesting that CIRP can modulate ER stress through TLR4 signaling pathway (52). Besides this, the CRIP release in sepsis is also reported to trigger adaptive immune system in the spleen by activating T-lymphocytes such as CD^4+^ and CD^8+^ T cells in a TLR4-depentent manner (28). Most importantly, the plasma level of CIRP of patients with sepsis is also correlated with the survival as the non-surviving patients have high level of serum CIRP than the survivors, suggesting that CIRP may act as a potent prognostic marker of sepsis in human (17). Altogether, it can be ruled out that CIRP plays acts as a mediator in organ dysfunction during sepsis by amplifying inflammation in immune cells and damaging vascular EC in all vital organs.

### 5.2 Lung injury

Acute lung injury (ALI)/acute respiratory distress syndrome (ARDS) is a complex clinical syndrome. eCIRP, as a type of DAMP, triggers inflammation in various inflammatory conditions including inflammatory lung injury (16, 68, 69). eCIRP can promote ALI by activating macrophages (16), neutrophils (57), pneumocytes (57), and pulmonary vascular endothelial cells (25).

A recent study (25) related to CIRP has shown that administering exogenous CIRP to healthy mice causes lung injury through vascular leakage, neutrophil infiltration, local production of pro-inflammatory cytokines, and activating the NLRP3 inflammasome in lung vascular endothelial cells. During the past years, a fair amount of studies have investigated the potential role of eCIRP in different models of inflammatory lung injury such as ALI caused by various diseases (52), chronic obstructive pulmonary disease (COPD) (70, 71), and pulmonary fibrosis (72). Recently, CIRP’s elevated expression was found in the bronchi in patients suffering from chronic obstructive pulmonary diseases and corresponding *in-vitro* study demonstrated that CIRP induces expression of inflammatory cytokines and mucin in human airway epithelial cells through activation of activating ERK and TLR4/NF-κB signaling pathway (70, 73). The important pro-inflammatory role of eCIRP suggests that targeting eCIRP may have a therapeutic potential in controlling inflammatory lung injury.

Lately, a study (74) has revealed the possible regulation pathway of eCIRP in ALI. It was found that the expression of CIRP increased in lung tissues of the LPS-induced ALI/ARDS mice and inhibited the polarization of M2 macrophages and increased the inflammatory response. eCIRP-neutralizing antibodies attenuated the M1 phenotype and enhanced the M2 phenotype in macrophages. eCIRP controls inflammation by regulating the phenotypic changes in macrophages. Inhibition of eCIRP was able to attenuate local and systemic inflammation like AP-associated acute lung injury (APALI) (68).

### 5.3 Neuroinflammation

Neuroinflammation is a disorder observed in the central nervous system (CNS) in response to infection, toxic metabolites, trauma, or autoimmune stimuli. Recent research (18) has revealed that CIRP has a considerable effect on neuroinflammation.

Alcohol, as an exogenous stimulant, affects the CNS and causes memory loss.Some researchers believe that eCIRP might be a key player in the relationship between alcohol and Alzheimer’s disease (AD) (31). Besides, both alcohol (31) and cerebral ischemia (19) resulted in the expression of eCIRP in microglial cells as well as its release. CIRP is a potential novel mediator of alcohol-induced brain inflammation, leading to local inflammation and neuronal cell damage *via* the TLR4 pathway (75). A recent study (59) reported that eCIRP activated neurotoxic cyclin-dependent kinase-5 (Cdk5)/p25 through the induction of the IL-6Rα/STAT3 pathway in neurons, and C23 subsided the eCIRP-induced increase in neuronal STAT3 phosphorylation and p25 level. Additionally, CIRP may activate TLR4 signaling and further induce NF-κB activation (75). In a mouse model of stroke induced by middle cerebral artery occlusion (MCAO)in CIRP deficient mice (19), TNF-α induction and microglial activation were significantly reduced, and the volume of cerebral infarction was attenuated after MCAO.

### 5.4 Ischemia-reperfusion Injury

Ischemia-reperfusion (I/R) injury is defined as the impaired tissue function after ischemia (76), which can occur in multiple organs causing organ damage and failure. CIRP has been shown to be an important factor involved in the regulation of I/R injury, and that it plays vital roles in the pathways of the liver (20), kidney (21, 22), and intestinal I/R injury (23, 24).

Following ischemic tissue damage and subsequent reperfusion, endogenous DAMPs, which are normally intracellular, are released extracellularly and trigger sterile inflammation (77). M3, an eCIRP-derived peptide (62), can inhibit TREM-1 by protecting mice against intestinal I/R injury and showed good therapeutic effects after reperfusion. The severity of organ injury was mitigated, serum levels of systemic inflammation markers such as IL-6, and TNF-α were reduced and the inflammation in the intestinal tissue itself was also improved.

Interestingly, recent evidence has established a causal link between CIRP and ALI during intestinal I/R injuries. In an adult male CIRP knock-out (CIRP ^-/-^) mouse model, pro-inflammatory cytokine, myeloperoxidase, and apoptotic cells were significantly lower than in C57BL/6J wild-type mice, which led to decreased lung injury (24). In another parallel experiment (23), the intraperitoneal injection of C23, 60 minutes after the ischemic insult, could mitigate the systemic inflammation, extent of injury and the ALI of intestinal I/R mice.

According to the results from an adult male C57BL/6J mouse model of hepatic I/R, circulating levels of CIRP were increased and the treatment of an anti-CIRP antibody reduced the inflammatory storm and cellular damage in the liver and significantly inhibited neutrophil infiltration into the liver (20).

There is also some evidence that CIRP may affect renal injury after I/R. The deficiency of CIRP reduces renal injury after renal I/R by reducing inflammation and oxidative stress (21). A study (22) showed that the expressions of kidney injury molecule-1 and neutrophil gelatinase-associated lipocalin were significantly decreased in C23-treated mice.

### 5.5 Idiopathic pulmonary fibrosis

Pulmonary fibrosis is a devastating sequela of many chronic inflammatory diseases characterized by a progressive decline in lung volume capacity and high mortality. eCIRP induces pro-inflammatory cytokines and differentially-expressed pathways in lung fibroblasts in a TLR4-dependent manner, and the accessory pathways MD2 and Myd88 are involved in the induction of the inflammatory phenotype (54). Furthermore, CIRP is involved in the regulation of pulmonary fibrosis, as eCIRP can directly activate and induce inflammatory phenotype fibroblasts (IPF) in the lung. In a recent research with bleomycin-induced pulmonary fibrosis in a mouse model, C23 alleviated pulmonary fibrosis and molecular markers of fibrosis were ameliorated in TLR4^-/-^mice (54), suggesting that eCIRP acts as a key a key promoter of PF, and blockage eCIRP with C23 can significantly attenuate this inflammatory PF.

## 6 Inhibitors of CIRP

### 6.1 Polypeptides

Focusing on the previously shown mechanisms of action of eCIRP, there are also some studies on novel therapeutic drugs. C23, an oligopeptide derived from the human CIRP protein, binds to the CIRP receptor with high affinity (16). Studies have shown that treatment with C23 decreased systemic inflammation in adult or neonatal sepsis mouse models (78, 79). Although C23 is the best polypeptide known thus block CIRP from its interaction, there exist many more inhibitors as well. C23 stands out among several short sequences derived from CIRP with a higher affinity for TLR4-MD2 receptor complexes compared to CIRP in terms of binding activity (16). As of today, C23 has been shown to act as a protective agent (22) against tissue damage resulting from hemorrhagic shock (78), sepsis (29), and intestinal ischemia-reperfusion (23) by inhibition of CIRP-induced inflammatory response. Remarkably, C23 has a far greater affinity to the TLR4-MD2 receptor complex than LPS and HMGB1, suggesting that C23 derived from CIRP may be a potential therapeutic approach for tissue and organ damage caused by cytokine storm.

M3 is another interesting CIRP-derived polypeptide with slight differences compared to C23. M3 acts on TREM-1, a receptor for eCIRP. During hepatic I/R, the binding of eCIRP to TREM-1 increases the number of inflammatory cells in the liver leading to increased tissue damage therein. M3 treatment, on the other hand, had an increased effect on the survival rate of mice after hepatic I/R (60). When M3 competes with TREM-1, it also blocks the eCIRP signaling pathway in the heart (80) and kidney (81). M3 exerts protective effects against sepsis-induced myocardial injury and acute kidney injury after renal I/R by inhibiting the eCIRP/TREM-1 interaction (81).

Similarly, pre-treating with M3, a human eCIRP-derived ligand-dependent 7-amino acid (aa) peptide, acts as an antagonist of TREM-1 (56), and LP17, a TREM-1 decoy peptide (82), significantly decreased the production of IL-6 and CXCL2 in the cuboidal type II cells (ATII) (57). MicroRNAs (miRNAs) have already been identified in the extracellular space and are involved in different physiological or pathological processes (83). Recent research also identified a novel interaction between miR-130b-3p and eCIRP *via* the TLR4 pathway. miRNA 130b-3p (84) serves as a new endogenous inhibitor of eCIRP-mediated inflammatory responses.

### 6.2 Natural products

Due to their anti-inflammatory properties, some natural products have been shown to be as effective as synthetic drugs in treating various diseases and ailments. There has been a lot of evidence that natural products and their derivatives can help reduce, inflammation, inflammatory cell infiltration, inflammatory signaling, and release of DAMPs *in-vitro* and *in-vivo* (85). In addition, because of their low toxicity and high safety profiles, they are usually the subject of a great deal of attention for clinical and therapeutic interventions.

For example, Emodin (EMO), which is an anthraquinone, has numerous pharmacological effects, particularly as a potent anti-inflammatory phytochemical. Pancreatic and lung tissues as well as the serum were shown to have high levels of CIRP in a rat model of acute pancreatitis, which was found to be associated with the formation of the NLRP3 inflammasome in alveolar macrophages and infiltration of neutrophils (25, 68). Intriguingly, EMO reverses these effects in rats thereby minimizes damage to the pancreas and lungs. In addition, EMO had a comparable pharmacological inhibitory action to C23 in rat alveolar macrophages, inhibiting the production of inflammatory mediators in the presence of CIRP. On such a basis, EMO could be a potential CIRP antagonist (68).

Another natural agent, Luteolin (LUT), has been also shown to inhibit the synthesis of CIRP. Lut has significant anti-inflammatory and antioxidant properties (86). Research suggests that Lut can reduce organ damage caused by I/R and endotoxemia by blocking the HMGB1 (a well-known DAMP) signaling pathway, indicating that Lut and DAMPs may have a unique functional relationship (87). Zhang et al. (88) showed that Lut treatment in neonatal mice with sepsis reduces the expression of CIRP mRNA and protein and attenuates lung injury. Lut also decreases CIRP formation in peritoneal macrophages following LPS administration. In contrast to EMO, the protective effect of Lut on neonatal mouse macrophages can be attributed to the reduction in the production of HIF-1 (89) and the NLRP3 (90) inflammasome, as indicated by *in vitro* experiments.

In the inflammatory response, endogenous CIRP-mediated signaling pathways cause damage to multiple organs. Antagonizing endogenous CIRP can help prevent I/R or sepsis-induced lung, kidney, heart, liver, and intestinal injury. By blocking CIRP-mediated signaling pathways, C23 and M3 effectively prevent organ damage. This is done through their effects on different receptors. Further investigations on whether natural products such as Luteolin (88) and Emodin (68) could act directly on the CIRP protein and its downstream receptor or by affecting the transcription of upstream genes need to be investigated in depth.

## 7 Exosomes as drug carriers in CIRP targeting-based therapies

Delivery systems use specialized carriers to facilitate the aggregation of drugs in their target tissues or cells in order to increase their efficacy and usage (91). Combining targeted medications with nanocarriers is one of the most important means of increasing the effectiveness of targeted therapeutics (92–94). Exosomes generated from intracellular membranes (95) have many benefits over conventional nanocarriers, including good biocompatibility and targeting (96), easy modification of the membrane surface (97), and the capacity to traverse the blood-brain barrier and placental barrier (98). As a new means of drug delivery, exosomes are gaining attention (99).

Exosome-based targeted drug delivery systems have been summarized in several studies (100). Naturally occurring exosomes can be used as carriers for a wide range of therapeutic agents such as natural products (101–104), synthetic pharmaceuticals (105), nucleic acid drugs (106, 107), and protein-based peptides or proteins (108). Exosomes can penetrate biological barriers that regular nanomaterials cannot, and they exhibit good efficacy. The exosome-carried CIRP brings a more severe inflammatory response to the disease while providing us with a potential therapeutic strategy (48, 109). Using exosomes that have been artificially modified may be a way to reduce the eCIRP-induced inflammatory response. On one hand, adding peptides or proteins to the surface of exosomes inhibits the activation of downstream signaling pathways by targeting inflammatory cell surface receptors (110, 111) and on the other hand, exosomes directly loaded with natural products through incubation (108), electroporation (112), sonication (113), and transfection exert anti-inflammatory effects. In conclusion, these methods may address the low water solubility and bioavailability of natural compounds (114). Moreover, the drug loading and modification (115) of EVs may enhance the targeting ability of the drug and prevent its rapid oxidation.

## 8 Conclusions

Since the first description of CIRP two decades ago, the physiopathological and biological roles of CIRP have been extensively studied. Although we have gained significant achievements in unraveling the role of CIRP in diseased etiology and pathogenesis; however, there are still many unanswered questions, for instance, what is the role of CIRP in chronic inflammatory diseases such as obesity, diabetes and other kinds of diseases. It has been proved that iCIRP participates in cell proliferation, cell survival, apoptosis, and circadian rhythm, telomere maintenance, and carcinoma progression through regulating mRNA stability as an RNA chaperone (28).

Whereas little is known about the function of exosome-carried CIRP and its role in inflammation. Here we summarized former and recent studies on the roles of eCIRP, especially exosome-derived CIRP, in inflammatory disease. One of the most notable recent developments is that eCIRP has been shown to play an important part in various types of ALI through participating in different inflammatory pathways. The discovery of eCIRP’s role as an important DAMP has led to a new field in inflammatory disease research, while many knowledge gaps still exist and need to be addressed. As a stabilizing RNA-binding protein, eCIRP is pro-inflammatory in most inflammatory conditions but also reduces inflammation by unknown mechanisms and pathways. On one hand, it remains to be investigated in other cell types such as B lymphocytes and NK cells since current studies have only focused on eCIRPs role in macrophages, neutrophils, and T cell biology. On the other hand, only macrophages have been identified as the source of exosomal CIRP, while other cell types as sources of exosomal CIRP remain to be studied.

Both *in-vivo* and *in-vitro* evidence support the notion that eCIRP can be considered as a novel target for the research and development of innovative drugs for the prevention or treatment of inflammatory diseases. Future studies should be aimed at clarifying the pivotal roles eCIRP plays in other potential pathways of inflammatory diseases in the hope that more therapeutic targets can be identified. Peptides and natural products targeting exosomal CIRP may facilitate the clinical translation of these new therapeutic strategies against inflammation.

The proinflammatory role and the plausible protective effects of CIRP blockage using neutralizing antibody or proteins/peptides in sepsis and other inflammatory diseases suggested a pivotal role of CIRP in inflammation-related diseases and has offered a strong basis for the exploration of therapeutic potential of neutralizing antibody or peptide in tackling various inflammatory diseases. Therefore, elucidating the role of CIRP in these diseases will have a significant impact on our understanding of physiopathological of diseases and may provide the rationale for the design of novel therapeutics.

## Author contributions

Conceptualization, CX, HC; writing—original draft preparation, JH, YZ, PG, TD, HW, ST, YL, QY, BH and GZ. All authors have edited the published version of the manuscript.

## Glossary

**Table:**

AD	Alzheimer's disease
ALI	acute lung injury
ARDS	acute respiratory distress syndrome
ATII	alveolar epithelial type II
Cdk5	cyclin-dependent kinase 5
CIRP	cold-inducible RNA-binding protein
CK2	Casein kinase 2
CNS	central nervous system
COPD	chronic obstructive pulmonary disease
DAMPs	damage-associated molecular patterns
eCIRP	extracellular cold-inducible RNA-binding protein
EMO	emodin
ER	endoplasmic reticulum
EV	extracellular vesicles
GSK3β	glycogen synthase kinase 3β
HMGB1	high mobility group box 1
I/R	ischemia-reperfusion
ICAM-1	intercellular adhesion molecule-1
iCIRP	intracellular cold-inducible RNA-binding protein
ILs	interleukins
ILVs	intraluminal vesicles
iNOS	inducible nitric oxide synthase
IPF	inflammatory phenotype fibroblasts
JAM-C	junctional adhesion molecule-C
LAMP	lysosome-associated membrane protein
LPS	lipopolysaccharide
LUT	luteolin
MCAO	middle cerebral artery occlusion
MD2	myeloid differentiation 2
mtDNA	mitochondrial DNA
MVBs	multi-vesicle bodies
MyD88	myeloid differentiation factor 88
NE	neutrophil elastase
NETs	neutrophil extracellular traps
NF-κB	nuclear factor-κB
NLRP3	NOD-like receptor protein 3
NLRs	NOD-like receptors
PAD4	peptidylarginine deiminase 4
RBP	RNA-binding protein
RRM	RNA-recognition motif
rTEM	reverse transendothelial migration
SAP	severe acute pancreatitis
SGs	granules
STAT3	signal transducer and activator of transcription 3
STING	stimulator of IFN genes
Syt	synaptotagmin
TfR	transferrin receptor
TIA-1	T-cell intracellular antigen-1
TLRs	Toll-like receptors
TNF	tumor necrosis factor
TREM-1	triggering receptor expressed on myeloid cells-1
UV	ultraviolet
