# Supplementary material for: Exosome-derived CIRP: An amplifier of inflammatory diseases
Source: Front Immunol. 2023 Feb 14;14:1066721. doi: 10.3389/fimmu.2023.1066721 (PMC9971932; doi:10.3389/fimmu.2023.1066721)
Supplement: Supplementary file 1 [file Table_1.docx]

Supplementary Table 1: Biological Roles and Functions of CIRP as a Proinflammatory Mediator of Different Diseases.

| Biological Roles and Functions | Possible Pathway, Mechanism or Implication | Model | References |
| --- | --- | --- | --- |
| Stimulates Inflammation in Sepsis and Hemorrhagic Shock | Binding and Interaction with TLR4/MD2 complex present of antigen processing cells (APCs) resulting into stimulation and release of TNFα and IL-6 | Human Serum, Rodent Models of Sepsis and Hemorrhagic Shock | [1, 2] |
| Activates splenic T-cells during sepsis, contributing to T-cell dysregulation | Binds TLR4/MD2 complex on CD4^+^ and CD8^+^ T-cells to induce activation and Th1 hyperinflammatory response | Mice with Induced Sepsis | [3] |
| Role in Synovial Inflammation in Patients with Rheumatoid Arthritis | CIRP as a marker for chronic synovial inflammation | Human Serum and Synovial Fluid | [4] |
| Lung Injury, Inflammation and Damage | Activation of TLR4/NFκB pathway of Inflammation and Tissue Damage | Human Bronchial Biopsies, Bronchial Epithelial Cells | [5, 6] |
| Activates caspase-1, IL-1β and induced pyroptosis; Activation of Lung Endothelial Cells Leading to Cell Damage | Assembly of the Nlrp3 inflammasome | Mouse Lung Vascular Endothelial Cells (MLVEC), WT Mice | [7, 8] |
| Induction of Transcription IL-1β, IL-8 and TNFα | Increases Iκκ phosphorylation and activating inflammation through NFκB pathway | Neonatal Foreskin Fibroblasts | [9] |
| Induction of Apoptosis in Neurons and Cardio | Positively regulates NFκB activation | Mouse Models of Neuroinflammation | [10] |
| Wound Healing | Decreases TNFα during the initial (inflammatory) phase of wound healing | Wild-type and CIRP Knockout Mice | [11] |
| Stimulation of NLRP3 Inflammasome in Human Neutrophils | Uric acid-induced mature IL-1β induction | Neutrophils | [12] |
| Liver Glycolysis | Activation of anti-apoptotic protein (Bcl-2), increased the level of protein kinase B (AKT) phosphorylation, and activation of the AKT-signaling pathway. | C57BL/6 Mice | [13] |
| Chronic Airway Inflammation Diseases | Increased expression of inflammatory cytokines and mucin in human airway epithelial cells by activation of ERK and NF-κB signaling pathway | Human Airway Epithelial Cells | [5, 14] |
